# SOX9 interacts with FOXC1 to activate MYC and regulate CDK7 inhibitor sensitivity in triple-negative breast cancer

**DOI:** 10.1038/s41389-020-0232-1

**Published:** 2020-05-12

**Authors:** Lin Tang, Juan Jin, Kun Xu, Xin Wang, Jinhai Tang, Xiaoxiang Guan

**Affiliations:** 1 Department of Medical Oncology, Jinling Hospital, Medical School of Nanjing University, Nanjing, 210002 China; 2 Department of Oncology, Fudan University Shanghai Cancer Center, Shanghai, 200032 China; 3 Department of Oncology, The First Affiliated Hospital of Nanjing Medical University, Nanjing, 210029 China; 4 Department of General Surgery, The First Affiliated Hospital of Nanjing Medical University, Nanjing, 210029 China

**Keywords:** Breast cancer, Predictive markers

## Abstract

Triple-negative breast cancer (TNBC) is particularly sensitive to cyclin-dependent kinase 7 inhibitor, THZ1, compared to hormone receptor (HR)+ breast cancer, but our data found that different TNBC cell lines had a wide range of IC_50_ values of THZ1, suggesting a possible heterogeneity in sensitivity to THZ1 in TNBC. To seek potential biomarkers of THZ1 sensitivity, we re-analyzed the mRNAs profile in breast cancer cells treated with THZ1 from the previous study and demonstrated that elevated expression of SOX9 was significantly associated with the sensitivity of THZ1 in TNBC. We also verified that SOX9 expression promoted cell proliferation, migration, stemness, and predicted poor prognosis. Moreover, based on the tissue array of 278 patients and over 900 samples from TCGA data, we found that SOX9 expression was significantly higher in TNBC than HR+ breast cancers. Furthermore, ChIP-sequencing indicated that SOX9 binding to enhancer near transcription factor FOXC1, was remarkably inhibited by THZ1. And we also demonstrated that SOX9 and FOXC1 interacted with each other, which might co-operate and co-regulate the MYC signaling pathway in TNBC. Mechanistically, SOX9 may sensitize TNBC cells to THZ1, in a FOXC1-related manner, suggesting that SOX9 could be as a predictive factor of THZ1.

## Introduction

Triple-negative breast cancer (TNBC) is a particularly aggressive subtype of breast cancer, which is characterized by high genetic complexity and lacks the distinct driver oncogenes^1^. Because of the tumor heterogeneity of TNBC, the development of targeted therapies has been limited for a long time^2^. Surprisingly, a recent research identifies that TNBC is selectively sensitive to THZ1, a newly developed CDK7 inhibitor^3^. Recent studies have found that besides the regulation of cell cycle, CDK7 is also essential in promoting transcription processes. CDK7 inhibitor can strongly diminish the transcription of a cluster of particular genes, which are overexpressed in TNBC but not in hormone receptor (HR)+ breast cancer cells containing putative oncogenes, promoting TNBC tumorigenicity and the disease development^3^. Moreover, an excessive number of genes in this cluster are associated with super-enhancers (SEs)^4,5^. It is commonly accepted that enhancers are regions of non-coding DNA, which mediate the transcription of adjacent genes, serving as a cis-regulatory element^6^. SEs are a subset of the enhancers with a pronounced effect on recruiting transcription factors, cofactors, and chromatin regulators to drive the abundant expression of some significant oncogenes, such as MYC, CCND2, and EGFR, in various tumors. CDK7 inhibitor is expected to control TNBC cell proliferation by inhibiting the super-enhancer-associated oncogenes^3^.

Although TNBC have showed an extreme sensitivity to THZ1 compared to HR+ breast cancer, different responses to the CDK7 inhibitor still exist among different TNBC cell lines shown by us and Wang and coworkers^3^. Therefore, to identify the factors that predict against the THZ1 response in TNBC may help with a better selection of TNBC patients for the treatment. Previous studies found that THZ1 elicited selective effects mainly by hampering highly active transcription and preferentially induced selective inactivation of super-enhancer-associated oncogenes^4,5,7,8^. If some oncogenic transcription factors are associated with SEs, malignant potency of the transcription factors will be amplified, which can trigger numerous and deregulate transcription processes in tumors. So, we focused on the super-enhancer-associated transcription factors and demonstrated that SOX9, which is a super-enhancer-associated transcription factor in TNBC cells, predicted the sensitivity of TNBC to THZ1. Most of the published studies reported that SOX9 as an oncogenic protein was overexpressed in various solid tumors, inducing cancer cell growth, proliferation, migration, and invasion^9–14^. Moreover, co-expression of exogenous Slug and Sox9 increased the tumorigenic and metastatic abilities of breast cancer cells and was associated with poor outcomes in breast cancer patients^11^. In the present study, we demonstrated that high SOX9 expression was associated with malignant phenotype in both TNBC cells and TNBC patients. However, the exact mechanisms underlying SOX9 functions during tumor formation and development largely remain unclear until now. Here, we are the first to demonstrate the roles of SOX9 in TNBC and the possible mechanisms of its predictive role in the THZ1 sensitivity.

## Results

### SOX9 predicted sensitivity to THZ1 in TNBC cells

**Fig. 1:**
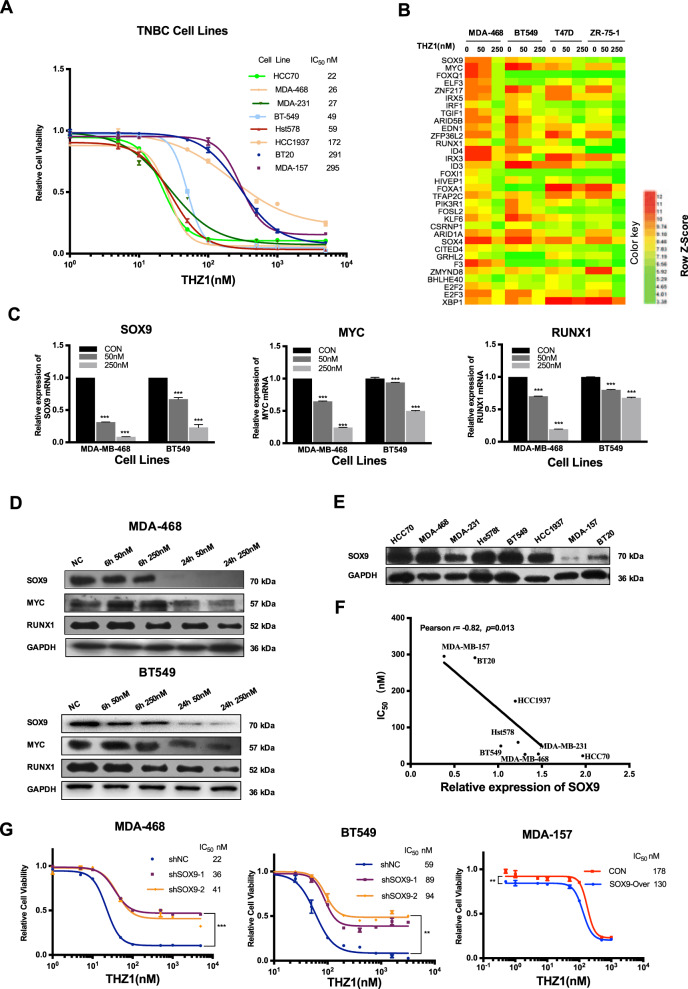
SOX9 enhanced the sensitivity of TNBC cells to THZ1. **a** Dose–response curves of 8 TNBC cell lines treated with indicated concentrations of THZ1 for 72 h were analyzed by CCK8 assay. (*n* = 3, mean ± SD). **b** Heatmap based on data from Wang et al. research^3^ represented microarray data of mRNA levels of super-enhancer-associated transcription factors, which were defined by super-enhancer data in Wang et al. study and PANTHER Classification System, in BT549, MDA-468, T47D, and ZR-75-1 breast cancer cells treated with THZ1 at the indicated concentrations for 6 h. **c** MDA-468 and BT549 were treated with 50 nM or 250 nM of THZ1 for 24 h. After treatment, qRT-PCR was performed to detect SOX9, MYC, and RUNX1 mRNA expression. (*n* = 3, mean ± SD, ***P < 0.001). **d** MDA-468 and BT549 were treated with 50 nM or 250 nM of THZ1 for 6 h or 24 h. After treatment, western blot was performed to detect SOX9, MYC, and RUNX1 protein expression. **e** Baseline levels of SOX9 expression in eight TNBC cell lines were determined by western blot. **f** Pearson correlation between gene expression of SOX9 and IC_50_ values of THZ1 in eight TNBC cell lines was shown. (*n* = 8, *r* = −0.82, *P* = 0.013). **g** MDA-468 and BT549 with knockdown of SOX9 and MDA-157 with overexpression of SOX9 were treated with the indicated concentrations of THZ1 for 72 h. Cell viability was analyzed by the CCK8 assay. (*n* = 3, mean ± SD, ***P < 0.001, **P < 0.01).

We demonstrated that THZ1 treatment indeed showed strong cytotoxicity in TNBC cell lines, consistent with a previously published study^3^, but we also observed that some TNBC cell lines, such as BT-20 and MDA-157, were not sensitive to THZ1 (Fig. 1a). Notably, the most insensitive cell lines were with IC_50_ values averaging almost ten times higher than those of sensitive ones (Fig. 1a), which hint that there are probably some factors responsible for the THZ1 efficacy in TNBC cells. We analyzed the gene expression values of super-enhancer-associated transcription factors based on the microarray data of two TNBC cell lines and two HR+ breast cancer cell lines treated with THZ1 from the previously published study^3^ (Fig. 1b). The ideal predictive factor is identified as elevated in TNBC cells compared to HR+ breast cancer cells and highly inhibited by the THZ1 treatment. So, we chose SOX9, RUNX1, and MYC for further investigations and showed that THZ1 treatment decreased the expression of SOX9, RUNX1, and MYC at both mRNA and protein levels (Fig. 1c, d).

Because SOX9 was inhibited more significantly than the other two factors upon THZ1 treatment in a dose- and time-dependent manner (Fig. 1d), we focused further on SOX9 that has not been evaluated deeply in TNBC. Moreover, in a panel of TNBC cell lines with varying levels of SOX9 expression, SOX9 expression levels were negative with IC_50_ values, and SOX9-amplified cells had a high sensitivity to THZ1, indicating that SOX9 would be a predictive factor for the THZ1 sensitivity (Fig. 1e, f). Next, we silenced SOX9 in MDA-468 and BT549 but overexpressed SOX9 in MDA-157 that had a relatively low baseline expression level of SOX9. We found that SOX9 knockdown increased the IC_50_ values in MDA-468 and BT549, while ectopic expression of SOX9 in MDA-157 sensitized cells to THZ1 (Fig. 1g). Together, these results showed that the SOX9 expression level was positively associated with the sensitivity to THZ1 in TNBC cells.

### SOX9 facilitated malignant properties in TNBC cells

**Fig. 2:**
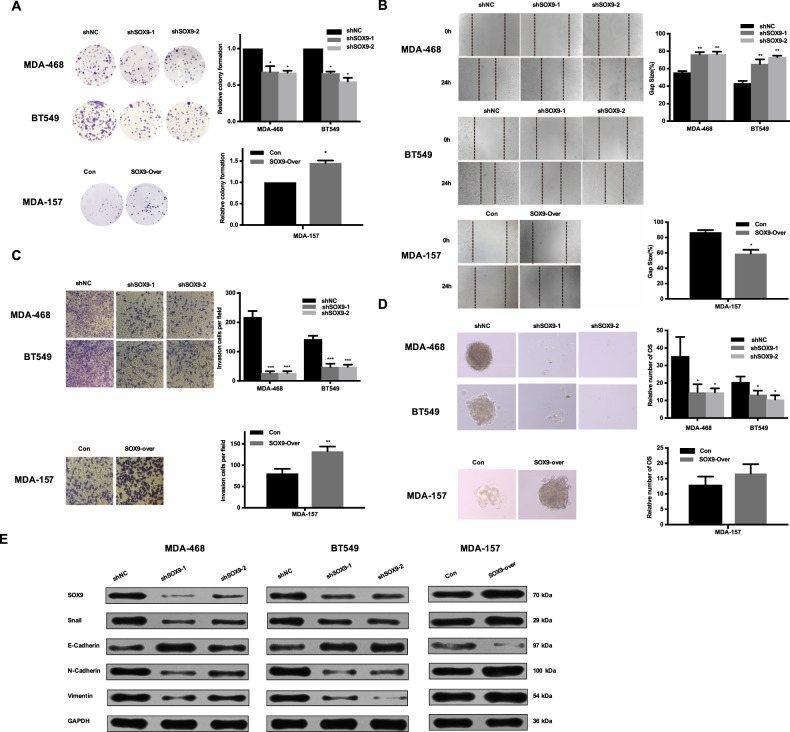
SOX9 promoted cell proliferation, cell migration and sustained breast cancer initiation, and EMT potential in TNBC. **a** Colony-forming images in MDA-468 and BT549 after SOX9 knockdown and quantitative data were shown. (*n* = 3, mean ± SD, *P < 0.05). **b** Respective images and quantitative data of the wound-healing assays in MDA-468 and BT549 after SOX9 deletion and in MDA-157 with overexpressed SOX9 were presented. (*n* = 3, mean ± SD, *P < 0.05, **P < 0.01). **c** Transwell assays results in MDA-468 and BT549 with SOX9 knockdown and in MDA-157 after SOX9 overexpression and quantitative data of the migration ability were indicated. (*n* = 3, mean ± SD, **P < 0.01, ***P < 0.001). **d** Respective images of the tumorsphere formation in MDA-468 and BT549 after SOX9 knockdown and in MDA-157 after SOX9 overexpression and quantitative data of the tumorsphere number were shown. (*n* = 3, mean ± SD, *P < 0.05). **e** Western blot analysis detected E-cadherin, N-cadherin, Snail, and Vimentin protein levels in MDA-468 and BT549 cells after SOX9 knockdown and in MDA-157 after SOX9 overexpression.

Although numerous studies have investigated the roles of SOX9 in cancers of the prostate and colorectum^10,12,14^, few investigated molecular functions of SOX9 in breast cancer. As shown in Supplementary Fig. S1, the cell proliferation, colony formation and migration ability of TNBC cells treated with THZ1 were significantly decreased, compared to the control group. To adequately address the mechanisms underlying the functional association between SOX9 and CDK7 inhibitor, we investigated the biological functions of SOX9 in TNBC cells. Colony formation assay showed that SOX9 silence distinctly inhibited cell proliferation, while SOX9 overexpression promoted cell proliferation (Fig. 2a). We also investigated the effect of SOX9 on cell migration in TNBC cells and showed that SOX9 inhibition weakened the migration potential of MDA-468 and BT549. In contrast, the forced overexpression of SOX9 promoted cell migration in MDA-157 (Fig. 2b, c).

Because several studies have identified SOX9 as an important stem cell marker, we tested the effect of SOX9 on tumorsphere formation^11^. SOX9 knockdown suppressed tumor-initiating ability with less tumorsphere formation compared to the control group. However, a statistic difference was not observed between MDA-157 and MDA-157 with SOX9 overexpression (Fig. 2d). Previous studies showed that epithelial-mesenchymal transition (EMT) progresses supported the stemness of cancer cells and regulated cell migration ability^15^. As shown in Fig. 2e, knockdown of SOX9 significantly increased the expression levels of the epithelial marker E-cadherin but decreased expression levels of the mesenchymal markers N-cadherin, Vimentin, and Snail. Furthermore, overexpression of SOX9 induced N-cadherin, Vimentin, and Snail expression upregulation and E-cadherin loss. This suggested that the SOX9 had a critical role in causing aggressive EMT features. In conclusion, these experiments indicated that SOX9 was a critical regulator that facilitated malignant properties in TNBC cells and that targeting SOX9 would be a potent strategy in TNBC treatment.

### TNBC patients with high levels of SOX9 expression had a poor prognosis

**Fig. 3:**
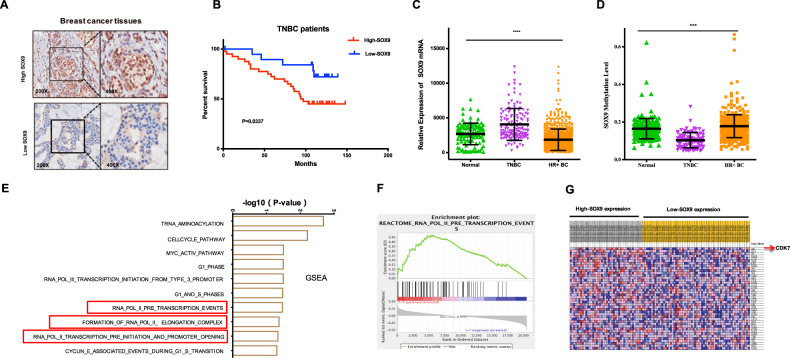
SOX9 was associated with poor prognosis in TNBC patients. **a** Representative immunohistochemical staining images showed SOX9 high- and low-expression in breast cancer patient tissues. **b** Kaplan–Meier curve showed OS in high and low SOX9-expressing tumor tissues in TNBC patients (*P* = 0.0337). **c** SOX9 mRNA levels in primary TNBC tumor tissues, HR+ breast cancer tissues, and normal adjacent tissues from TCGA database were as shown. (***P < 0.001). **d** SOX9 gene methylation levels of primary TNBC tumor tissues, HR+ breast cancer tissues and normal adjacent tissues from TCGA database were analyzed. (***P < 0.001). **e** GSEA indicated the top ten signaling pathways in SOX9 high TNBC tumors compared to SOX9 low tumors determined by −log (P). **f** GSEA result of the RNA-pol-II-pre-transcription-events signaling pathway comparing SOX9 high and SOX9 low TNBC samples in the TCGA data set. **g** Heatmaps represented the expression of components in this pathway among patients with high versus low levels of SOX9 expression.

The previous study found that the SOX9 staining was medium positive in tumor tissues of breast cancer, relative to other types of tumors, but exact relationship between SOX9 and clinical characteristics of breast cancer patients has not been shown^10,16^. To evaluate the clinical roles of SOX9, we performed immunohistochemical (IHC) staining against SOX9 in two tissue microarrays (Fig. 3a). A total of 278 cases are included in the final analysis, containing 58 TNBC patients. In addition, we detected 143 (51.43%) of 278 cases with high SOX9 expression and 135 (48.57%) of 278 cases with low SOX9 expression. For correlations of SOX9 expression with different clinicopathological factors of breast cancer patients, patients with high SOX9 expression levels seemed to be related to advanced tumor stages (Table 1). More importantly, SOX9 was inversely associated with estrogen receptor (ER) and progesterone receptor (PR) status, suggesting that SOX9 might be highly positive in HR- breast cancer (Table 1). TNBC patients with low SOX9 expression would have a longer overall survival (OS) based on our tissue microarray data (Fig. 3b). And the *p*-value in univariate and multivariate Cox proportional hazards model was 0.034 and 0.024, respectively.

**Table 1:** Relationship between expression of SOX9 and clinicopathologic characteristics of breast cancer patients.

Variables	Sox9 low (135)		Sox9 high (143)		*P*-value
	No.	%	No.	%	
*Age*					0.474
<53 years	70	51.85%	68	47.55%	
≥53 years	65	48.15%	75	52.45%	
*Pathology grade*^a^					0.014
Grade 1	22	16.42%	11	7.69%	
Grade 2	95	70.90%	99	69.23%	
Grade 3	17	12.69%	33	23.08%	
*T stage*^b^					0.038
1	40	30.08%	46	32.17%	
2	92	69.17%	88	61.54%	
3	1	0.75%	9	6.29%	
*Nodal status*^c^					0.748
Negative	56	44.09%	62	45.58%	
1–3 positive nodes	35	27.56%	36	26.47%	
4–9 positive nodes	31	24.40%	29	21.32%	
≥10 positive nodes	5	3.94%	9	6.62%	
*ER status*^d^					<0.0001
Positive	103	81.75%	71	50.35%	
Negative	23	18.25%	70	49.65%	
*PR status*^e^					<0.0001
Positive	88	68.75%	60	42.55%	
Negative	40	31.25%	81	57.45%	
*Her2 status*^f^					0.525
Positive	29	24.17%	36	27.69%	
Negative	91	75.83%	94	72.31%	
*Outcome*^g^					0.085
Death	29	21.64%	44	30.77%	
Survival	105	78.36%	99	69.23%	
*Subtypes*^h^					
Luminal A	75	64.1%	57	40.43%	0.001
Luminal B	20	17.09%	22	15.60%	
HER2-positive	6	5.13%	20	14.18%	
TNBC	16	13.68%	42	29.79%	

^a^1 sample of pathology grade status of Sox9 low expression group was missed.

^b^2 samples of T stage status of Sox9 low and high expression group were missed, respectively.

^c^8 and 7 samples of nodal status of Sox9 low and high expression group were missed, respectively.

^d^7 and 2 samples of ER status of Sox9 low and high expression group were missed, respectively.

^e^7 and 2 samples of PR status of Sox9 low and high expression group were missed, respectively.

^f^15 and 13 samples of HER2 status of Sox9 low and high expression group were missed, respectively.

^g^1 sample of outcome status of Sox9 low expression group was missed, respectively.

^h^18 and 2 samples of subtype status of Sox9 low and high expression group were missed, respectively.

To expand our observation, we utilized available mRNA expression and epigenetic data of SOX9 in breast cancer patients collected from The Cancer Genome Atlas (TCGA) database. TNBC showed an increased SOX9 mRNA expression and a decreased DNA methylation at the SOX9 locus compared to normal tissue (Fig. 3c, d). Intriguingly, the results also revealed that HR+ breast cancer had a decreased SOX9 expression and an increased SOX9 methylation relative to normal tissue or TNBC subtype, indicating that SOX9 tended to be epigenetically and transcriptionally silenced in HR+ breast cancers, similar to our IHC results (Fig. 3c, d; Table 1). These results demonstrated that SOX9 was a key regulator in TNBC and that its association with survival was consistent with the oncogenic functions of SOX9 in TNBC cells. To address SOX9-related signal pathways comprehensively, we performed gene set enrichment analysis (GSEA) in TNBC samples with high and low SOX9 expression from the TCGA data. Notably, the pathways associated with transcription regulation, cell cycle, and MYC activation were among the top pathways (Fig. 3e). The RNA pol-II pre-transcription events pathway was enriched in TNBC with high expression of SOX9 (s-h) compared with that in TNBC with low expression of SOX9 (s-l). Notably, CDK7 ranked as the top one gene in the RNA pol-II pre-transcription events pathway, highlighting clinically a significant association between SOX9 and CDK7 (Fig. 3f, g). These results indicate that SOX9 had a powerful potential of promoting transcription progress in TNBC.

### THZ1 inhibited the binding of SOX9 on enhancers of super-enhancer-associated transcription factors

**Fig. 4:**
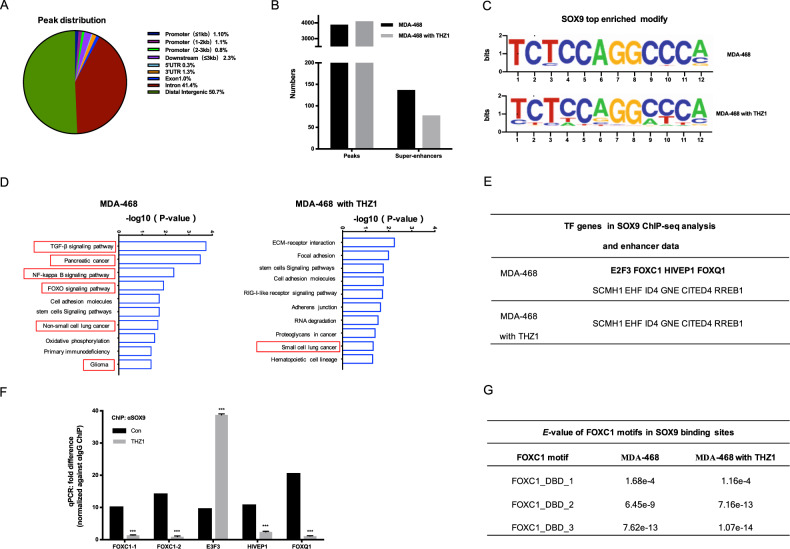
THZ1 inhibited the binding of SOX9 on enhancers of SE-associated transcription factor FOXC1. **a** Peak distribution of SOX9 ChIP-seq data in MDA-468; **b** Analysis based on data of super-enhancer sequences in MDA-468^3^ and ChIP-seq data of SOX9 binding sites showed that SOX9 bound on decreased super-enhancers in MDA-468 treated with 25 nM THZ1 for 24 h compared to the control cells. **c** The motifs enriched in SOX9 binding sites in MDA-468 and MDA-468 treated with 25 nM THZ1 for 24 h were shown. **d** Gene ontology (GO) analysis of genes associated with SOX9 binding sites determined by −log (P). **e** Following 25 nM THZ1 treatment for 24 h, ChIP-seq found that the bindings of SOX9 on several enhancers near super-enhancer-associated transcription factors disappeared. **f** ChIP-seq results were shown at the top. ChIP-qPCR was used to detect the binding of SOX9 with putative enhancer elements near FOXC1, E2F3, HIVEP1, and FOXQ1, based on results in (**e**). The PCR targets were named as the transcription factor gene near them. qPCR experiments were shown at the bottom. (***P < 0.001) **g** *E*-value of FOXC1 motifs in SOX9 binding sites in MDA-468 and MDA-468 treated with THZ1. *E*-value is the lowest p-value of any spacing of the secondary motif times the number of secondary motifs. It estimates the expected number of random secondary motifs that would have the observed minimum *p*-value or less.

To gain an insight into the regulation of SOX9-specific transcription and effect of THZ1 on the SOX9-regulated transcription, we performed chromatin immunoprecipitation with high-throughput sequencing (ChIP-seq) in MDA-468 following treatment with THZ1. ChIP-seq experiment detected 3,906 peaks in MDA-468 cells. The majority of SOX9-binding sites were enriched in distal intergenic and intron regions (Fig. 4a). Strikingly, after 24 h treatment with THZ1, by comparing the sequences of SOX9-binding peaks with all super-enhancer sequences in MDA-468, we found that THZ1 inhibited the binding of SOX9 on SEs at the condition that the total peak numbers did not decrease (Fig. 4b). As shown for the top sequence motifs(TCTCCAGGCCCA) in SOX9 binding regions, which were not similar to the canonical SOX9 motif (AGAACAATGG)^16^, THZ1 did not change the binding motif of SOX9 in MDA-468 too much (Fig. 4c). Furthermore, the gene ontology (GO) analysis indicated that genes associated with SOX9-binding sites (within 100 kb upstream and downstream of each gene) were related to a variety of oncogenic pathways, indicating the potentially important oncogenic function of SOX9 as a transcription factor. Strikingly, after the THZ1 treatment, the pathways associated with tumor phenotypes were decreased, suggesting an anti-tumor effect of THZ1 (Fig. 4d). Our further analysis focused on SE associated transcription factor genes, and the results showed that THZ1 attenuated the binding of SOX9 on enhancers of these transcription factor genes (Fig. 4e). Next, we performed ChIP-quantitative (q) PCR on five informative genes as shown in Fig. 4f, using an antibody directed against SOX9. The results showed that THZ1 inhibited the binding of SOX9 to enhancers near FOXC1, E2F3, and FOXQ1, suggesting that THZ1 inhibits the transcription network regulated by SOX9. Next, we chose FOXC1 for further study because: (1) THZ1 inhibited the binding of SOX9 on two enhancers near FOXC1, which reflected a relatively greater effect on FOXC1 transcription and expression, further ChIP-seq analysis revealed that SOX9 binding sites harbored FOXC1 motifs (Fig. 4g); and (2) numerous documents have indicated that overexpression of the transcription factor FOXC1 is an important and special biomarker of TNBC^17^.

### SOX9 and FOXC1 interacted with each other and promoted TNBC

**Fig. 5:**
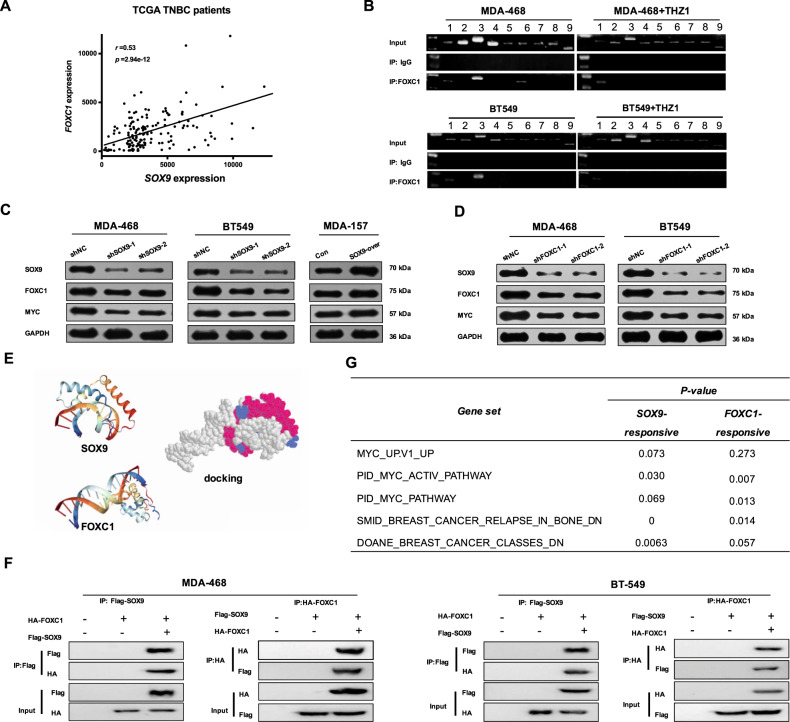
SOX9 and FOXC1 interacted with each other and promoted TNBC. **a** Expression levels of SOX9 and FOXC1 in TCGA microarray data of 137 TNBC tumors. Each dot represented one tumor. Linear trend line and Pearson correlation coefficient (*r* and *p*) were shown. **b** ChIP assay was performed using FOXC1 antibody in TNBC cells. FOXC1 immunoprecipitated fraction was subjected to PCR amplification with the nine primers sets of SOX9 promoters. **c** Western blots detected the expression level of SOX9, FOXC1, and MYC after SOX9 knockdown in TNBC cells. **d** The expression levels of FOXC1, SOX9, and MYC after FOXC1 knockdown were determined by western blot in TNBC cells. **e** Molecular docking of SOX9 and FOXC1. **f** Co-immunoprecipitation assay was performed to investigate SOX9 and FOXC1 protein interaction in TNBC cells. **g** GSEA results indicated that SOX9- and FOXC1-resposive genes were enriched of the proto-oncogenic MYC and breast cancer development pathways.

Finally, we examined the correlation between SOX9 and FOXC1 expression in TNBC samples in the TCGA database, by using mRNA expression microarray data. Surprisingly, among the all genes, the expression of FOXC1 was the most highly associated with that of SOX9 (*r* value = 0.53, *p* value = 2.94e−12, Matlab correlation function; Fig. 5a, Supplementary Table S1). The ChIP-qPCR results indicated that FOXC1 directly bound to the SOX9 promoter, and, moreover, the binding was reduced by the THZ1 treatment (Fig. 5b). Besides, SOX9 knockdown significantly reduced FOXC1 protein levels in TNBC cells, while SOX9 overexpression increased FOXC1 expression (Fig. 5c). Interestingly, FOXC1 knockdown also markedly diminished SOX9 protein levels in TNBC cells. Both SOX9 and FOXC1 knockdown reduced the protein expression of MYC in TNBC cells, suggesting the regulation of SOX9 and FOXC1 on the important oncogene (Fig. 5d). Having observed that positive association between SOX9 and FOXC1 in TNBC cells, we first predicted the 3D structures of SOX9 and FOXC1 and performed a protein–protein docking analysis with SWISS-MODEL. The result showed that SOX9 could interact with FOXC1 and was illustrated with Phyre2 tools, as shown in Fig. 5e. To investigate whether SOX9 and FOXC1 physically interact with each other. The MDA-468 and BT549 cells were transfected with Flag-tagged SOX9 (SOX9-flag) or/and HA-tagged FOXC1 (FOXC1-HA) plasmids for 24 h to conduct co-immunoprecipitation assays. Further results indicated that the protein complex immunoprecipitated by anti-Flag Ab was also detected by anti-HA Ab, which confirmed that SOX9 physically interacted with FOXC1 (Fig. 5f). These results suggest that SOX9 and FOXC1 might co-operate and co-regulate the same genes in some conditions. To identify the roles of SOX9/FOXC1 complex-mediated genes in clinic setting, we performed GSEA on SOX9- and FOXC1-responsive genes, which were identified as the genes positively associated with high SOX9 and FOXC1 expression, respectively. The results indicated significant enrichment for the previously identified MYC signaling pathway and pathways related to breast cancer development (Fig. 5g).

**Fig. 6:**
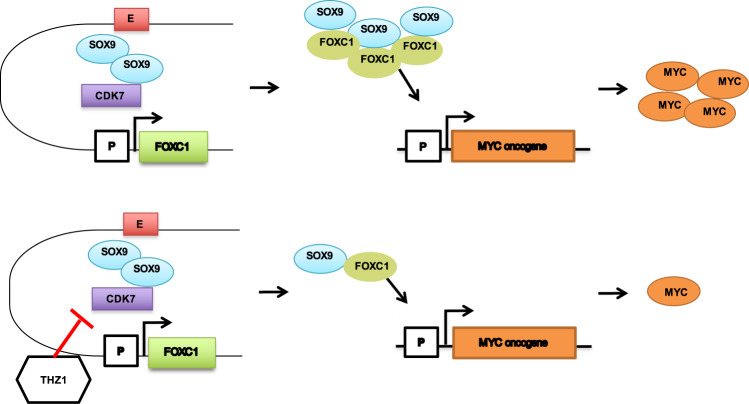
Scheme of the SOX9-FOXC1 model in TNBC. SOX9 activates FOXC1 transcription by binding its enhancers. Then, SOX9 and FOXC1 collaborate to activate an oncogenic gene regulatory program. THZ1 can inhibit the binding of SOX9 on enhancers near FOXC1 by inhibiting CDK7.

Conclusively, we found that a CDK7 inhibitor, THZ1, inhibited the expression of super-enhancer-associated oncogenic SOX9 and the transcription activation of SOX9 to FOXC1, a vital TNBC-special gene. We also found that SOX9 and FOXC1 interacted with each other and regulated the important oncogene MYC, but the positive loop could be blocked by THZ1 (Fig. 6).

## Discussion

Since the CDK7 inhibitor, THZ1, is developed, it has attracted considerable attention. It shows the potent cytotoxicity in various tumors, particularly for some malignant tumors lacking of effective treatments, such as TNBC and small cell lung cancer^3,18^. However, the divergent effects of THZ1 still were observed among different TNBC cell lines (Fig. 1a). It is commonly accepted that THZ1 can significantly disrupt the transcription of a set of oncogenes regulated by SEs^3,5^. A recent study found that amplified super-enhancer-associated MYCN oncogene, upregulating the active transcriptional program, sensitized cancer cells to inhibition of CDK7 in neuroblastoma^7,18^. In this study, we demonstrated that super-enhancer-associated transcription factor SOX9 contributed to the sensitivity of TNBC cells to THZ1. Previous studies have reported that SOX9 is highly expressed in prostate cancer and colorectal cancer and that SOX9 overexpression accelerated the formation and development of tumors^12,13^. Here, we presented functional and clinical data of broadly critical roles for SOX9 as an oncogenic factor in TNBC. We believe that SOX9 inhibition will be an effective measure to control TNBC. More interestingly, although TNBC cells showed exceptional sensitivity to THZ1 compared to HR+ breast cancer^3^, we have shown that SOX9 mRNA and methylation level were strikingly higher and lower, respectively, in TNBC than HR+ breast cancer, which might explain the different sensitivity between TNBC and HR+ breast cancer.

As a transcription factor, SOX9 is supposed to activate transcription network, reflected by GSEA results, probably by regulating a series of transcription factors. Our ChIP-seq data demonstrated that THZ1 really blocked the binding of SOX9 on SEs, thus providing a compelling evidence for impairment of transcription after THZ1 treatment. Further ChIP-qPCR verified that THZ1 reduced the binding of SOX9 on enhancers near several super-enhancer-associated transcription factor, especially including FOXC1. FOXC1 was also an important transcription factor and was identified as a critical molecular marker in TNBC, contributing to malignant phenotype of TNBC^17^. We have shown that SOX9 promoted FOXC1 expression by activating its enhancers by ChIP-qPCR. Disruption of the binding by THZ1 might be the mechanism of THZ1 action in TNBC cells. Both SOX9 and FOXC1 deletion could lower MYC protein level, which was consistent with our speculation that both SOX9 and FOXC1, although limited in scope, might mediate MYC protein translation and/or stability. Future studies are needed to detect this possibility and to clarify the potential underlying mechanism.

In conclusion, we believe that TNBC patients with high SOX9 expression are vulnerable to the THZ1 treatment. Our integrative analysis of SOX9 functions in TNBC and its relationship with FOXC1 has identified that SOX9 is a novel TNBC oncogene. More importantly, interventions of the SOX9-FOXC1-MYC pathway genes may provide potential strategies for the TNBC treatment.

## Materials and methods

### Cell Culture, Cell viability assay, and colony-forming assays

MDA-468, BT549 and MDA-157 cell lines were obtained from the Chinese Academy of Science Committee Type Culture Collection Cell Bank (Shanghai, China). Authenticity of these cell lines was done by Chinese Academy of Science Committee Type Culture Collection Cell Bank before purchase by STR DNA typing methodology. MDA-157 and BT549 cells were cultured in RPMI 1640 medium supplemented with 10% fetal bovine serum (FBS), MDA-468 cells were grown in DMEM supplemented with 10% FBS. All cell lines were cultured at 37 °C in a 5% CO_2_ atmosphere. For cell viability analysis, cells were seeded in 96-well plates at 4–6 × 10^3^ cells per well. After 24 h, the cells were exposed to different concentrations of agents and after 72 h exposure cell survival was measured with the Cell Counting Kit-8 in accordance with the recommended guideline (KeyGEN Biotech, Nanjing, China). Colony formation assays were performed as previously described^19^.

### Antibodies and agents

Antibodies against FOXC1 (#8758), rabbit IgG (#7074), mouse IgG (#7076) and GAPDH (#5174) were obtained from Cell Signaling Technologies; Anti-SOX9 (ab3697), anti-MYC (ab32072), anti-RUNX1 (ab138377) anti-Snail (ab53519), anti-E-cadherin (ab133597), anti-N-cadherin (ab76011) and anti-Vimentin (ab92547) were from Abcam; Antibodies were used at the manufacturer’s recommended dilutions. THZ1 was obtained from MedChem Express and diluted in DMSO.

### Clinical samples

Breast cancer tissue section containing HBre-Duc140Sur-03 (140 cases) and HBre-Duc140Sur-01 (140 cases) were provided by Outdo Biotech (Shanghai, China). Histological parameters were performed in accordance with the criteria of the World Health Organization. Pathologic staging was determined by the current International Union against Cancer tumor-lymph node metastasis classication. The follow-up information of breast cancer patients were as follows: the operation time of these patients was from January 2001 to August 2004 and the final follow-up was July 2013, thus the follow-up range was 9–12.5 years. Among this follow-up time, 73 out of 278 patients died of breast cancer with a median OS time of 62 months (2–147 months), and 205 patients were still alive with a median OS time of 123 months (40–150 months). The clinical information of breast cancer patients were shown in Table 1. A total of 278 cases are included in the final analysis except for 2 cases missed clinical staging information.

### Immunohistochemistry

Immunohistochemistry of breast tumor tissue samples were performed as previously described^20^. SOX9-positive cell staining is localized in the nucleus. The two times scoring method based on the positive ratio and staining intensity of tumor cells was used^21^: the number of positive tumor cells < 5% was 0, 5–25% was 1, 26–50% was 2, and >50% was 3. Dyeing intensity: 0 point for non-coloring; 1 point for light yellow; 2 points for dark yellow or brownish yellow; 3 points for tan particles. Multiplying the two for its final result: 0 to 1 is negative (−); 2 –3 is weakly positive (+); 4–5 is positive (++); ≥6 is strong positive (+++).

### Plasmid and lentivirus transfection

The human full-length cDNA of SOX9 were cloned into the pFLAG-CMV expression vector (Hanbio Biotechnology; Shanghai, China) and verified by sequence analysis before transfection. MDA-157 cells were transfected with an empty vector or pFLAG-CMV-SOX9 by Lipofectamine 2000 (Invitrogen, USA) following selection by 2 μg/ml puromycin (Gibco) according to the manufacturer’s protocol. The lentiviruses transfected with plasmid pGLV containing shRNA sequences were produced by Genepharma (Shanghai, China). The shRNA sequences were as follows: shSOX9-1: CCACCTTCACCTACATGAA, shSOX9-2: CAGCGAACGCACATCAAGA, shFOXC1-1: ACAAGAAGATCACCCTGAA, shFOXC1-2: AGAGGATCGGCTTGAACAA and shNC: AGTGCACGTGCATGTCCTA. Cells were transfected with lentiviral vectors following standard procedures from Genepharma. After transfection 72 h, selection was done using puromycin for 48 h and passaged further.

### RNA isolation and quantitative RT-PCR

Total RNA was extracted from cultured cells using Trizol (Invitrogen, USA) according to the manufacturer’s protocol. For qRT-PCR analysis, the cDNA was synthesized using a PrimeScriptTM RT Master Mix (Perfect Real Time) Kit (RR036A, Takara, China), followed by PCR using Power SYBR Green PCR Master Mix (Life Technology, USA). GAPDH was used as an internal control. The primer sequences were as follows: Sox9-forward: AGCGAACGCACATCAAGAC and reverse: CTGTAGGCGATCTGTTGGGG; RUNX1-forward: TCTTCACAAACCCACCGCAA and reverse: CTGCCGATGTCTTCGAGGTTC; MYC-forward: CGTCTCCACACATCAGCACAA and reverse: CACTGTCCAACTTGACCCTCTTG; GAPDH-forward: AAATCAAGTGGGGCGATGCTG and reverse: GCAGAGATGATGACCCTTTTG. The relative expression of SOX9, FOXC1 and MYC was determined using the 2−ΔΔCt method.

### Western Blot

After the indicated treatment, the cells were washed twice with PBS and lysed in RIPA buffer supplemented with protease and phosphatase inhibitors. Protein concentrations were determined with the BCA kit (All kits from KeyGEN Biotech, Nanjing, China). Western blot analysis was conducted using the above-mentioned antibodies, then the subsequent steps were performed as previously described^19,20^.

### ChIP-Seq and ChIP-PCR

ChIP-Seq was performed using anti-SOX9 antibody as previously described^22^. Immunoprecipitated DNA was purified after phenol extraction and genomic libraries were sequenced to 50 bp for an Illumina Hi-Seq 2000 (Illumina). ChIP-seq narrow peaks were called against the input using MACS2 software (version 2.0.9, *p*-value threshold = 1e−5). All the motifs were searching using the peak summits by extending 250 bp along the both ends. Motif discover using the HOMER packages. And we used the HOMER’s peaks annotation function to annotate out called peaks. The following sequences of primers were designed for SOX9 promoter in ChIP-PCR analysis. 9 primer sets of about 300 bp per primer were synthesized for the SOX9 promoter region (Supplementary Table S2). The sequences amplified by primer set 1 is the fastest from the transcription start site.

### TCGA data analysis

mRNA data and the methylation data of TCGA samples were downloaded through the TCGA data portal (https://cancergenome.nih.gov) and DiseaseMeth (http://www.bio-bigdata.com/diseasemeth/index.html), respectively. SOX9 high tumors were defined as those among the top 75% of tumors with positive z-scores in the TCGA data. In contrast, SOX9 low tumors were defined as those among the bottom 75% of tumors with negative z scores in the TCGA data.

### Co-immunoprecipitation assays

To investigate SOX9 and FOXC1 protein interactions, co-immunoprecipitation was performed in MDA-468 and BT-549. Cells were transfected with the indicated plasmids for 24 h, and nuclear extracts were prepared as described above. The supernatant was incubated with anti-HA agarose or anti-FLAG agarose (Sigma) overnight at 4 °C with constant agitation. These samples were further analyzed by immunoblotting. After centrifugation at 5000 × *g* for 3 min at 4 °C, the precipitates were then washed thrice with lysis buffer and boiled for 5 min in 2 × SDS sample buffer. Samples were analyzed by SDS-PAGE and transferred to PVDF membrane. For the detection of tag proteins, the whole-cell lysates were analyzed using immunoblotting technique with the appropriate antibody (Sigma). The transfection of empty vector alone into cells acted as a control.

### Molecular docking

The protein 3D structure of SOX9 and FOXC1 were predicted using local prediction bioinformatics tools, Phyre2 and SWISS-MODEL. Protein docking simulation was conducted with the ZDOCK server (http://zdock.umassmed.edu/). The local server is used to process and beautify the docking data file.

### Statistical analysis

All statistical tests were conducted with GraphPad Prism version 6.0. Data were analyzed using a Student’s *t*-test and One-way ANOVA. Data are presented as mean ± SD of three independent experiments unless stated otherwise. A *P* value of <0.05 was considered statistically significant. *P < 0.05, or **P < 0.01 or ***P < 0.001.

## Supplementary information

Supplement figure legends

Supplement Table S1

Supplement Table S2

Figure S1
