# Supplementary material for: SOX9 interacts with FOXC1 to activate MYC and regulate CDK7 inhibitor sensitivity in triple-negative breast cancer
Source: Oncogenesis. 2020 May 12;9(5):47. doi: 10.1038/s41389-020-0232-1 (PMC7217837; doi:10.1038/s41389-020-0232-1)
Supplement: Supplementary file 1 — Supplement figure legends [file 41389_2020_232_MOESM1_ESM.docx]

**SUPPLEMENTAL FIGURE LEGENDS**

**Supplementary Fig. S1 THZ1 inhibit cell proliferation and migration in TNBC.**

1. Colony-forming images in MDA-468 and BT549 after THZ1 treatment and quantitative data were shown. (n = 3, mean ± SD, *P < 0.05, **P < 0.01).
2. Respective images and quantitative data of the wound-healing assays in MDA-468 and BT549 after THZ1 treatment. (n = 3, mean ± SD, *P < 0.05, **P < 0.01).
3. Transwell assays results in MDA-468 and BT549 after THZ1 treatment and quantitative data of the migration ability were indicated. (n = 3, mean ± SD, **P <0.01, ***P < 0.001).

**Supplementary Table S1 Correlation of genes with SOX9 in clinical TNBC samples from TCGA database.**

**Supplementary Table S2 The following sequences of primers were designed for SOX9 promoter in ChIP-PCR analysis.**
