# Supplementary material for: SOX9 interacts with FOXC1 to activate MYC and regulate CDK7 inhibitor sensitivity in triple-negative breast cancer
Source: Oncogenesis. 2020 May 12;9(5):47. doi: 10.1038/s41389-020-0232-1 (PMC7217837; doi:10.1038/s41389-020-0232-1)
Supplement: Supplementary file 2 — Supplement Table S1 [file 41389_2020_232_MOESM2_ESM.docx]

| Gene Name | r value | P value |
| --- | --- | --- |
| FOXC1 | 0.52590821 | 2.94E-12 |
| WIPI1 | 0.49218265 | 1.04E-10 |
| NDST3 | 0.43631048 | 1.72E-08 |
| LOC100133545 | 0.42570396 | 4.12E-08 |
| CCL28 | 0.42447054 | 4.55E-08 |
| RAB40B | 0.42254053 | 5.31E-08 |
| AMZ2 | 0.40971617 | 1.45E-07 |
| TNP1 | 0.40875609 | 1.56E-07 |
| PCDHB3 | 0.38607642 | 8.27E-07 |
| SUSD4 | 0.38296937 | 1.03E-06 |
| FBLN5 | 0.37255289 | 2.11E-06 |
| DEFB109P1B | 0.37187561 | 2.21E-06 |
| PDGFRA | 0.37001371 | 2.51E-06 |
| DMRTB1 | 0.36515817 | 3.47E-06 |
| GAPDH | 0.36102101 | 4.55E-06 |
| OR8K5 | 0.36073962 | 4.63E-06 |
| CYP2W1 | 0.35923883 | 5.11E-06 |
| IRX1 | 0.35531844 | 6.58E-06 |
| VWDE | 0.35236187 | 7.94E-06 |
| MST4 | 0.34829433 | 1.03E-05 |
| C4orf7 | 0.34731355 | 1.09E-05 |
| GPC5 | 0.34612312 | 1.17E-05 |
| FZD7 | 0.34478463 | 1.27E-05 |
| GMDS | 0.34268265 | 1.45E-05 |
| PTX3 | 0.33764376 | 1.97E-05 |
| FOXK2 | 0.33726965 | 2.02E-05 |
| TRDN | 0.33698901 | 2.05E-05 |
| SPACA3 | 0.33453663 | 2.37E-05 |
| SLC6A16 | 0.33445245 | 2.39E-05 |
| NUPL2 | 0.3342284 | 2.42E-05 |
| FGFRL1 | 0.33197214 | 2.76E-05 |
| WDR45L | 0.32979735 | 3.14E-05 |
| C6orf35 | 0.32755939 | 3.58E-05 |
| WBP2 | 0.32637418 | 3.83E-05 |
| HOMER2 | 0.32484019 | 4.19E-05 |
| TSPAN6 | 0.32448913 | 4.28E-05 |
| PCDHB9 | 0.31989358 | 5.56E-05 |
| C1GALT1 | 0.31780654 | 6.25E-05 |
| OR5T2 | 0.31547388 | 7.12E-05 |
| PSMD12 | 0.31329127 | 8.04E-05 |
| TIGD1 | 0.31297708 | 8.18E-05 |
| RWDD2B | 0.31279774 | 8.26E-05 |
| SUMO2 | 0.31260659 | 8.35E-05 |
| ELAVL1 | 0.31231758 | 8.49E-05 |
| RPS2P32 | 0.31024292 | 9.51E-05 |
| UCK2 | 0.31019421 | 9.54E-05 |
| LOC100270746 | 0.30960694 | 9.85E-05 |
| COX19 | 0.30841086 | 0.00010509 |
| SRP68 | 0.30815595 | 0.00010656 |
| CSNK1D | 0.30711175 | 0.00011276 |
| H3F3B | 0.30703848 | 0.00011321 |
| SNORA31 | 0.30550944 | 0.00012294 |
| TRIM47 | 0.30539737 | 0.00012369 |
| COG1 | 0.30459941 | 0.0001291 |
| BACE2 | 0.30413488 | 0.00013236 |
| LOC146880 | 0.30380631 | 0.0001347 |
| THADA | 0.30364141 | 0.0001359 |
| KLHL7 | 0.30349412 | 0.00013697 |
| BBOX1 | 0.30343794 | 0.00013738 |
| MGP | 0.30315572 | 0.00013947 |
| RASGEF1B | 0.30246666 | 0.00014468 |
| FN3KRP | 0.30227665 | 0.00014615 |
| P2RX2 | 0.30172449 | 0.0001505 |
| PCDHB10 | 0.30160955 | 0.00015142 |
| HEATR2 | 0.3010824 | 0.00015571 |
| ZNF750 | 0.30049158 | 0.00016065 |
| YWHAE | 0.3002545 | 0.00016268 |
| RASAL2 | 0.29934626 | 0.00017065 |
| LOC100302401 | 0.29923285 | 0.00017168 |
| KPNA2 | 0.29784514 | 0.00018464 |
| PRKRA | 0.29666428 | 0.00019638 |
| HMGCLL1 | 0.29645977 | 0.00019849 |
| OSBPL3 | 0.29473087 | 0.00021712 |
| DUSP23 | 0.29327346 | 0.00023408 |
| CRABP2 | 0.29240676 | 0.00024474 |
| MRPL18 | 0.29216555 | 0.00024778 |
| OR51I1 | 0.29196581 | 0.00025033 |
| NRN1 | 0.29105846 | 0.00026222 |
| NTN3 | 0.29068552 | 0.00026726 |
| H19 | 0.28959149 | 0.00028256 |
| PDE6D | 0.28822583 | 0.00030279 |
| PSMG1 | 0.28794012 | 0.00030719 |
| WDR75 | 0.28749204 | 0.00031421 |
| ZNF165 | 0.28556028 | 0.00034621 |
| OR8K3 | 0.28506427 | 0.0003549 |
| COL9A3 | 0.284902 | 0.00035779 |
| ECEL1 | 0.28476842 | 0.00036018 |
| SUMO4 | 0.28407638 | 0.00037281 |
| FN3K | 0.28333567 | 0.00038679 |
| RHBDF2 | 0.28313162 | 0.00039072 |
| PTH2R | 0.28246271 | 0.00040388 |
| PTMA | 0.28150578 | 0.00042342 |
| PCDHA6 | 0.28141911 | 0.00042523 |
| SERPINA3 | 0.28120567 | 0.00042972 |
| EFNA5 | 0.2811524 | 0.00043085 |
| TM4SF1 | 0.28017481 | 0.00045205 |
| WBP5 | 0.27951885 | 0.0004668 |
| MALL | 0.27873422 | 0.00048504 |
| HN1 | 0.27810272 | 0.00050019 |
| ZC3H15 | 0.27787726 | 0.00050571 |
| LPL | 0.2778216 | 0.00050708 |
| GABRP | 0.27718129 | 0.00052309 |
| FAM189B | 0.27555974 | 0.00056574 |
| TFB1M | 0.27548039 | 0.00056791 |
| PWRN1 | 0.27460853 | 0.00059223 |
| PDK3 | 0.27460389 | 0.00059237 |
| OR8J1 | 0.27419841 | 0.000604 |
| C6orf124 | 0.27385372 | 0.00061406 |
| KRTAP1-1 | 0.27372832 | 0.00061776 |
| SULT1C4 | 0.27319153 | 0.00063382 |
| GPR45 | 0.2731154 | 0.00063613 |
| TMEM200A | 0.27300532 | 0.00063948 |
| ZNF716 | 0.27148553 | 0.00068745 |
| SEPT9 | 0.27145187 | 0.00068855 |
| KIAA0114 | 0.2708025 | 0.00071007 |
| KRTAP5-10 | 0.27075449 | 0.00071169 |
| ARG2 | 0.27073114 | 0.00071247 |
| KRTCAP3 | 0.27052666 | 0.0007194 |
| FAM104A | 0.27034053 | 0.00072576 |
| KCTD13 | 0.26882719 | 0.0007794 |
| NANOS3 | 0.26803199 | 0.00080902 |
| PDGFA | 0.26780044 | 0.00081784 |
| FAM27C | 0.26713575 | 0.00084364 |
| KCNMB3 | 0.26708107 | 0.0008458 |
| C17orf95 | 0.26610959 | 0.00088495 |
| EVPL | 0.26578876 | 0.00089825 |
| FOXD1 | 0.26475347 | 0.0009424 |
| HEBP2 | 0.26379384 | 0.00098509 |
| DDA1 | 0.26322147 | 0.00101139 |
| RPL38 | 0.26244231 | 0.00104823 |
| SCGB3A1 | 0.26218151 | 0.00106084 |
| CMTM7 | 0.26157474 | 0.0010907 |
| C15orf2 | 0.26149339 | 0.00109476 |
| PRSS16 | 0.26107927 | 0.00111565 |
| ACYP2 | 0.26040805 | 0.00115028 |
| SLC6A14 | 0.26019571 | 0.00116144 |
| ATP5H | 0.26004003 | 0.00116969 |
| MRPL3 | 0.25987151 | 0.00117867 |
| LRRTM4 | 0.25985489 | 0.00117956 |
| PFN2 | 0.25982957 | 0.00118092 |
| MGST3 | 0.25912719 | 0.00121912 |
| H3F3A | 0.25892202 | 0.00123049 |
| SAP30BP | 0.25881946 | 0.00123621 |
| NR1D1 | 0.25868509 | 0.00124374 |
| CSN3 | 0.25768123 | 0.00130134 |
| ITGB8 | 0.25724669 | 0.00132702 |
| PVRL4 | 0.25722928 | 0.00132806 |
| GPT | 0.25689219 | 0.00134831 |
| PIK3CA | 0.25677466 | 0.00135544 |
| SUN1 | 0.2566463 | 0.00136327 |
| HYMAI | 0.25576178 | 0.00141831 |
| KCNMB2 | 0.2556594 | 0.00142481 |
| MPV17L2 | 0.25558586 | 0.0014295 |
| NHSL1 | 0.25525186 | 0.00145096 |
| KIF1A | 0.25497533 | 0.00146895 |
| FAM20C | 0.25447348 | 0.00150212 |
| ICT1 | 0.25408178 | 0.00152849 |
| FAM64A | 0.25394055 | 0.00153809 |
| LOC285796 | 0.25360949 | 0.00156083 |
| OR5T1 | 0.25287451 | 0.00161242 |
| CHRNB1 | 0.25238349 | 0.00164774 |
| CHERP | 0.25233164 | 0.00165151 |
| SFXN1 | 0.2523112 | 0.001653 |
| IFNGR2 | 0.25218438 | 0.00166226 |
| FRAS1 | 0.25213431 | 0.00166593 |
| SPINT1 | 0.25143117 | 0.00171826 |
| TRIM55 | 0.25133561 | 0.00172548 |
| HAX1 | 0.2510295 | 0.00174882 |
| TFCP2L1 | 0.25069797 | 0.00177441 |
| VRK2 | 0.25035794 | 0.00180102 |
| POU3F4 | 0.25003187 | 0.00182687 |
| MORF4L2 | 0.24994977 | 0.00183344 |
| TPI1 | 0.24977689 | 0.00184732 |
| ENPP6 | 0.24943238 | 0.00187529 |
| HPRT1 | 0.2478246 | 0.00201095 |
| TRO | 0.24766096 | 0.00202524 |
| ETV6 | 0.24675932 | 0.00210568 |
| KCNK5 | 0.24671545 | 0.00210967 |
| FAM165B | 0.24659135 | 0.00212098 |
| C3orf26 | 0.24648864 | 0.00213039 |
| SNORD1C | 0.24608925 | 0.00216733 |
| ACTG1 | 0.24604299 | 0.00217164 |
| MAGED1 | 0.24575864 | 0.00219834 |
| BHLHB9 | 0.24549317 | 0.00222354 |
| MED26 | 0.24506892 | 0.00226435 |
| C6orf115 | 0.24501936 | 0.00226916 |
| NPY | 0.24465484 | 0.00230483 |
| HAUS8 | 0.24454092 | 0.00231609 |
| C6orf15 | 0.24439257 | 0.00233081 |
| PFDN2 | 0.24385111 | 0.00238529 |
| TFG | 0.24381317 | 0.00238915 |
| C19orf73 | 0.24378697 | 0.00239182 |
| VWC2 | 0.24366556 | 0.00240423 |
| RAB3A | 0.24366009 | 0.00240478 |
| PPL | 0.24349036 | 0.00242223 |
| PAPSS1 | 0.24346892 | 0.00242444 |
| PCDHGA1 | 0.2432232 | 0.00244991 |
| RAD51L1 | 0.24316281 | 0.00245621 |
| ZDHHC14 | 0.24266658 | 0.00250852 |
| OTUB2 | 0.2425566 | 0.00252025 |
| TSPAN12 | 0.24227908 | 0.00255006 |
| C17orf80 | 0.24223099 | 0.00255526 |
| APP | 0.24198054 | 0.0025825 |
| NDUFA7 | 0.24193859 | 0.00258708 |
| WTAP | 0.24192147 | 0.00258896 |
| FKBP9 | 0.24169114 | 0.00261429 |
| SLC25A19 | 0.24127996 | 0.00266007 |
| LEPREL2 | 0.2411955 | 0.00266957 |
| KRTAP5-7 | 0.24115537 | 0.00267409 |
| ADORA2B | 0.24100584 | 0.002691 |
| LOC100190939 | 0.2401987 | 0.00278395 |
| LOC100134713 | 0.24008183 | 0.00279765 |
| PFDN6 | 0.23990626 | 0.00281834 |
| CLDN6 | 0.23986052 | 0.00282375 |
| CXCL16 | 0.23974251 | 0.00283776 |
| GPR161 | 0.23954499 | 0.00286135 |
| POU6F2 | 0.23933003 | 0.00288722 |
| RRAS2 | 0.23902676 | 0.00292409 |
| KRTAP5-1 | 0.23896643 | 0.00293147 |
| HDAC8 | 0.23878169 | 0.00295418 |
| C2orf64 | 0.23876193 | 0.00295662 |
| ANGPTL4 | 0.23846995 | 0.00299287 |
| CLIP4 | 0.23846585 | 0.00299339 |
| C2orf82 | 0.23845618 | 0.00299459 |
| C10orf11 | 0.23844496 | 0.002996 |
| LOC729234 | 0.23834958 | 0.00300794 |
| CNKSR1 | 0.2383129 | 0.00301254 |
| ZNF177 | 0.23824028 | 0.00302167 |
| CRIPT | 0.23805192 | 0.00304548 |
| TRIM65 | 0.23800589 | 0.00305132 |
| EIF2B2 | 0.23795006 | 0.00305842 |
| TUSC3 | 0.23786199 | 0.00306965 |
| C11orf49 | 0.2373132 | 0.00314047 |
| PLAGL1 | 0.23554434 | 0.0033789 |
| C19orf53 | 0.2346571 | 0.00350454 |
| GNL2 | 0.2345696 | 0.00351715 |
| RIPK4 | 0.23441185 | 0.00354 |
| LSM4 | 0.23440192 | 0.00354144 |
| LDOC1 | 0.23439692 | 0.00354217 |
| S100A1 | 0.23431628 | 0.00355391 |
| ATL2 | 0.23408639 | 0.00358756 |
| LOC550643 | 0.2331702 | 0.00372455 |
| EXOC7 | 0.23299094 | 0.0037519 |
| NARF | 0.23254589 | 0.00382058 |
| IRX2 | 0.23248736 | 0.00382969 |
| YIPF2 | 0.23228047 | 0.00386207 |
| UROC1 | 0.23205722 | 0.00389729 |
| FBN3 | 0.2319519 | 0.003914 |
| NUP37 | 0.2319359 | 0.00391654 |
| SYT14L | 0.23165781 | 0.00396101 |
| LOC84740 | 0.2314446 | 0.00399541 |
| C2orf70 | 0.23109933 | 0.00405168 |
| RNF175 | 0.2310714 | 0.00405626 |
| C6orf164 | 0.23086793 | 0.00408979 |
| SLC7A5 | 0.2307188 | 0.00411453 |
| ADI1 | 0.2303092 | 0.00418315 |
| BEYLA | 0.22976721 | 0.00427554 |
| RAVER1 | 0.22972618 | 0.0042826 |
| TIPRL | 0.22948195 | 0.00432489 |
| EIF4A3 | 0.22941029 | 0.00433737 |
| BIRC7 | 0.22940748 | 0.00433786 |
| DYRK4 | 0.22939639 | 0.00433979 |
| MBD5 | 0.22914667 | 0.00438355 |
| NOL11 | 0.22895586 | 0.00441726 |
| TOMM5 | 0.22807791 | 0.00457535 |
| MFSD6L | 0.22799974 | 0.00458967 |
| ORC4L | 0.22762868 | 0.00465819 |
| METTL5 | 0.22741352 | 0.00469834 |
| ZNF322B | 0.22733337 | 0.00471337 |
| FARSB | 0.22709929 | 0.00475753 |
| FAM200B | 0.22672437 | 0.00482903 |
| SLC26A4 | 0.22647639 | 0.00487685 |
| HSPA7 | 0.22639701 | 0.00489225 |
| DKK4 | 0.22616421 | 0.00493765 |
| SPATA7 | 0.22613659 | 0.00494306 |
| IGFL4 | 0.2261158 | 0.00494714 |
| BHLHE41 | 0.22605155 | 0.00495976 |
| BFAR | 0.22595814 | 0.00497816 |
| KRTAP5-9 | 0.22587933 | 0.00499373 |
| GOLGA6C | 0.22567199 | 0.0050349 |
| DAGLA | 0.22545254 | 0.00507881 |
| TMCO6 | 0.22531777 | 0.00510594 |
| ELOVL6 | 0.22522471 | 0.00512475 |
| LOC401397 | 0.2250954 | 0.00515099 |
| TEAD2 | 0.22506267 | 0.00515766 |
| ARMCX5 | 0.22502086 | 0.00516618 |
| PGAM2 | 0.2249553 | 0.00517956 |
| DEFB1 | 0.22491584 | 0.00518763 |
| CDK2AP1 | 0.22487629 | 0.00519573 |
| TNNI1 | 0.22476719 | 0.00521814 |
| RPP21 | 0.2247417 | 0.00522339 |
| RCC2 | 0.22461532 | 0.00524948 |
| FAM3D | 0.22456365 | 0.00526017 |
| SLC24A5 | 0.2243082 | 0.00531336 |
| KIF20A | 0.22430435 | 0.00531416 |
| CXorf51 | 0.22428122 | 0.005319 |
| MRPS30 | 0.22394196 | 0.00539044 |
| DBX1 | 0.22376082 | 0.00542894 |
| EIF5A | 0.22355123 | 0.00547379 |
| C1QTNF1 | 0.22339149 | 0.00550819 |
| OR10S1 | 0.22337382 | 0.00551201 |
| C19orf50 | 0.22311592 | 0.00556799 |
| LOC643008 | 0.22309153 | 0.00557332 |
| AIG1 | 0.22300558 | 0.0055921 |
| UBE2Q1 | 0.22290949 | 0.00561317 |
| C1RL | 0.22286639 | 0.00562265 |
| TCP1 | 0.22280718 | 0.00563569 |
| FOXD3 | 0.22257076 | 0.00568802 |
| LOC145474 | 0.22225403 | 0.0057588 |
| CXorf61 | 0.22222022 | 0.0057664 |
| PAPL | 0.22208768 | 0.00579629 |
| UST | 0.22163141 | 0.00590025 |
| CADM4 | 0.22115356 | 0.0060109 |
| ADAT2 | 0.22089054 | 0.00607259 |
| ATP2A1 | 0.2208814 | 0.00607474 |
| FAM171B | 0.2206237 | 0.00613575 |
| DYNLT1 | 0.22043909 | 0.00617979 |
| H1FNT | 0.22024503 | 0.00622639 |
| THAP4 | 0.21987587 | 0.0063159 |
| KARS | 0.2196243 | 0.00637756 |
| UBA52 | 0.21962076 | 0.00637843 |
| ULBP2 | 0.2195122 | 0.0064052 |
| ZPLD1 | 0.21949104 | 0.00641044 |
| C14orf70 | 0.21919205 | 0.00648475 |
| DKFZP686I15217 | 0.21916613 | 0.00649122 |
| COL27A1 | 0.21914801 | 0.00649576 |
| B4GALNT4 | 0.21893564 | 0.00654909 |
| SNORA76 | 0.21885343 | 0.00656983 |
| PRMT2 | 0.21883675 | 0.00657405 |
| KLHL26 | 0.21849729 | 0.00666039 |
| TPD52L1 | 0.21843297 | 0.00667687 |
| ILF3 | 0.21834152 | 0.00670035 |
| LOC100101938 | 0.21827669 | 0.00671704 |
| EDN3 | 0.21793852 | 0.00680472 |
| DPY19L2P1 | 0.21784686 | 0.00682866 |
| THUMPD2 | 0.21759114 | 0.00689584 |
| PPP1R7 | 0.21741743 | 0.00694181 |
| EHHADH | 0.21737705 | 0.00695254 |
| CMTM2 | 0.21732397 | 0.00696666 |
| VN1R2 | 0.21700491 | 0.00705208 |
| IQCG | 0.21661879 | 0.0071567 |
| MAST1 | 0.21644071 | 0.00720541 |
| HBXIP | 0.21642066 | 0.00721091 |
| GABRA5 | 0.21640514 | 0.00721518 |
| PLRG1 | 0.21627521 | 0.00725095 |
| PLXDC2 | 0.21625096 | 0.00725764 |
| SUPT7L | 0.21605191 | 0.00731279 |
| OR5AU1 | 0.21600411 | 0.00732609 |
| ZSWIM4 | 0.21576396 | 0.00739323 |
| DDX39 | 0.21565547 | 0.00742374 |
| NME4 | 0.21560671 | 0.00743749 |
| KHDRBS3 | 0.21558481 | 0.00744367 |
| HJURP | 0.21552776 | 0.0074598 |
| CLDN10 | 0.21515826 | 0.00756501 |
| OR2T11 | 0.21500889 | 0.00760791 |
| PAM | 0.21487647 | 0.00764612 |
| NR2C2AP | 0.21474964 | 0.00768288 |
| DNASE1L2 | 0.21461033 | 0.00772344 |
| MRPL39 | 0.21450722 | 0.00775358 |
| ITGB4 | 0.21439867 | 0.00778543 |
| NR1I3 | 0.2143483 | 0.00780024 |
| MKRN3 | 0.21434472 | 0.0078013 |
| GRIK2 | 0.2143366 | 0.00780369 |
| AFF2 | 0.21425948 | 0.00782643 |
| LRRN1 | 0.21409673 | 0.00787461 |
| SEPT2 | 0.21389298 | 0.00793531 |
| LYG1 | 0.21387854 | 0.00793963 |
| CLK2 | 0.21369885 | 0.00799353 |
| ARHGEF19 | 0.2136875 | 0.00799694 |
| TTLL13 | 0.21355964 | 0.0080355 |
| CLLU1OS | 0.21332314 | 0.00810727 |
| BTG3 | 0.2131861 | 0.00814911 |
| ACOX1 | 0.21315501 | 0.00815863 |
| TATDN3 | 0.212833 | 0.00825781 |
| ARMCX2 | 0.21247533 | 0.00836922 |
| NFIX | 0.21242033 | 0.00838647 |
| SCO1 | 0.21239639 | 0.00839399 |
| FOLR1 | 0.21227335 | 0.00843272 |
| KIAA0317 | 0.21218677 | 0.00846007 |
| LIPG | 0.21215098 | 0.0084714 |
| XDH | 0.21185801 | 0.00856465 |
| ARHGEF10 | 0.21176299 | 0.00859508 |
| GSK3A | 0.21172487 | 0.00860732 |
| KCNN4 | 0.21167101 | 0.00862464 |
| MT1F | 0.21150405 | 0.00867851 |
| CPAMD8 | 0.2114497 | 0.00869611 |
| CA6 | 0.21112411 | 0.00880222 |
| TRIM43 | 0.21109992 | 0.00881015 |
| LOC25845 | 0.21102477 | 0.00883482 |
| COPS7B | 0.21098313 | 0.00884852 |
| FAM27A | 0.21097028 | 0.00885275 |
| IER3 | 0.21093512 | 0.00886434 |
| CEP55 | 0.21068234 | 0.00894803 |
| SYCN | 0.21063381 | 0.00896417 |
| ST8SIA5 | 0.21060178 | 0.00897484 |
| RPL36A | 0.21056854 | 0.00898593 |
| B3GNT9 | 0.21044685 | 0.00902662 |
| DCD | 0.21027702 | 0.00908367 |
| KRTAP3-2 | 0.21022339 | 0.00910176 |
| HMGN5 | 0.2101989 | 0.00911002 |
| JPH1 | 0.21013337 | 0.00913218 |
| UBE2O | 0.20992192 | 0.009204 |
| MDFI | 0.20987244 | 0.00922088 |
| CSDA | 0.20975322 | 0.00926166 |
| PAK3 | 0.2097366 | 0.00926736 |
| FAM127B | 0.20972703 | 0.00927064 |
| DDX11L2 | 0.20961902 | 0.00930776 |
| FANCL | 0.20960592 | 0.00931227 |
| MRPL36 | 0.20952557 | 0.00933998 |
| CDK7 | 0.20943908 | 0.00936988 |
| GINS2 | 0.20925391 | 0.00943419 |
| OSMR | 0.20913488 | 0.00947574 |
| TGM5 | 0.20884379 | 0.00957802 |
| CAPN6 | 0.20877874 | 0.00960101 |
| RCOR2 | 0.20857675 | 0.0096727 |
| DLGAP1 | 0.20844841 | 0.0097185 |
| TAC3 | 0.20839955 | 0.00973599 |
| RIPK2 | 0.20838648 | 0.00974067 |
| CCT3 | 0.20838201 | 0.00974227 |
| S100A13 | 0.20832005 | 0.0097645 |
| NOS1 | 0.20827989 | 0.00977893 |
| FZD8 | 0.20824819 | 0.00979034 |
| PRDXDD1P | 0.20824584 | 0.00979118 |
| DSCR9 | 0.2082277 | 0.00979771 |
| ZMAT3 | 0.20813434 | 0.00983139 |
| TIGD2 | 0.2081178 | 0.00983737 |
| C5orf46 | 0.20810283 | 0.00984278 |
| COX10 | 0.20786156 | 0.00993037 |
| CFTR | 0.2078608 | 0.00993065 |
| LRP8 | 0.2078273 | 0.00994287 |
| PCDHGB5 | 0.20778851 | 0.00995703 |
| POLR3G | 0.20771212 | 0.00998497 |
| DPCD | 0.20759412 | 0.01002827 |
| FKRP | 0.20743913 | 0.0100854 |
| FTMT | 0.20735725 | 0.01011569 |
| LRFN1 | 0.20725159 | 0.0101549 |
| ITGB3BP | 0.2072385 | 0.01015977 |
| C14orf1 | 0.20723455 | 0.01016124 |
| MCTP2 | 0.20712845 | 0.01020077 |
| VNN1 | 0.20689281 | 0.01028904 |
| CHAC2 | 0.20674044 | 0.01034649 |
| ZNF85 | 0.20651315 | 0.0104327 |
| TIAF1 | 0.20641132 | 0.01047152 |
| VSNL1 | 0.20611019 | 0.01058709 |
| C6orf138 | 0.20608921 | 0.01059518 |
| C7orf36 | 0.20597909 | 0.01063776 |
| FAM100B | 0.20593961 | 0.01065305 |
| ARL16 | 0.20590442 | 0.01066671 |
| SSPN | 0.20588651 | 0.01067366 |
| C21orf121 | 0.20577048 | 0.01071881 |
| FLJ45244 | 0.20559633 | 0.0107869 |
| SH3YL1 | 0.20535286 | 0.01088272 |
| RGS2 | 0.20521651 | 0.0109367 |
| LGTN | 0.20505992 | 0.010999 |
| FAM36A | 0.2048854 | 0.01106879 |
| ERH | 0.20481914 | 0.01109539 |
| GTF2H5 | 0.20454557 | 0.01120582 |
| TOMM34 | 0.20451792 | 0.01121703 |
| ALDH18A1 | 0.20444043 | 0.01124851 |
| GRHL3 | 0.2043796 | 0.01127328 |
| LOC286002 | 0.20430511 | 0.01130367 |
| SMO | 0.20423621 | 0.01133185 |
| R3HDM1 | 0.20394263 | 0.0114526 |
| RRP1B | 0.20384113 | 0.01149461 |
| TSG1 | 0.20373596 | 0.01153829 |
| SYT8 | 0.20373513 | 0.01153863 |
| SERPINB2 | 0.20373276 | 0.01153962 |
| MMP7 | 0.20371021 | 0.011549 |
| SPATA12 | 0.20354843 | 0.01161652 |
| EIF3CL | 0.20351911 | 0.0116288 |
| LAD1 | 0.20348993 | 0.01164102 |
| CYCS | 0.20331395 | 0.011715 |
| CCDC58 | 0.20329786 | 0.01172178 |
| AVPI1 | 0.20323516 | 0.01174825 |
| UGP2 | 0.20290176 | 0.01188989 |
| EHBP1 | 0.20284967 | 0.01191215 |
| CD9 | 0.20273872 | 0.0119597 |
| FAM57A | 0.20257776 | 0.01202897 |
| ISM1 | 0.20245171 | 0.01208346 |
| PRKAR1B | 0.20239771 | 0.01210687 |
| NGEF | 0.20225668 | 0.01216819 |
| GTPBP3 | 0.2022468 | 0.0121725 |
| PPPDE2 | 0.20213706 | 0.01222043 |
| MMACHC | 0.20205681 | 0.01225557 |
| MRPL34 | 0.20175179 | 0.01238999 |
| SIPA1L1 | 0.20167736 | 0.01242299 |
| PIAS2 | 0.20167469 | 0.01242417 |
| DNAJC24 | 0.2016489 | 0.01243562 |
| DTNB | 0.20157309 | 0.01246933 |
| SERPINB10 | 0.20134081 | 0.01257314 |
| OLFM2 | 0.20129734 | 0.01259265 |
| C9orf40 | 0.20126726 | 0.01260616 |
| PRKAR1A | 0.20117539 | 0.01264753 |
| SNHG3 | 0.20114962 | 0.01265915 |
| MARS2 | 0.20110315 | 0.01268013 |
| BCAS2 | 0.20106896 | 0.01269558 |
| MPZL1 | 0.20106341 | 0.0126981 |
| SALL3 | 0.20105348 | 0.01270259 |
| LOC283050 | 0.20101293 | 0.01272095 |
| KIAA1211 | 0.20083946 | 0.01279977 |
| EIF3M | 0.20078301 | 0.01282551 |
| PROM1 | 0.20069222 | 0.01286701 |
| TAL2 | 0.20056005 | 0.01292762 |
| WRB | 0.20045639 | 0.01297534 |
| ORMDL1 | 0.20025436 | 0.01306878 |
| RCN1 | 0.20007705 | 0.01315127 |
