# Supplementary material for: SOX9 interacts with FOXC1 to activate MYC and regulate CDK7 inhibitor sensitivity in triple-negative breast cancer
Source: Oncogenesis. 2020 May 12;9(5):47. doi: 10.1038/s41389-020-0232-1 (PMC7217837; doi:10.1038/s41389-020-0232-1)
Supplement: Supplementary file 3 — Supplement Table S2 [file 41389_2020_232_MOESM3_ESM.docx]

| **Sequences of primers for SOX9 promoter (5' to 3')** | | |
| --- | --- | --- |
| **No.** | **Forward** | **Reverse** |
| **1** | TGTGTCTCCGCTCCCGG | ATTTCTGCAGGGGCCTCCTG |
| **2** | CGAATGGAGCCCCTGAATGG | TGGACTGCTTTGCTGCTGAG |
| **3** | TCAGCAGCAAAGCAGTCCA | AGGAGACTCGGAGACCATCG |
| **4** | AAATGTCTGCCCGATGGTCTC | CGGAAGAAGCCCGACATTTCT |
| **5** | GAAATGTCGGGCTTCTTCCG | GGAGGCATTGGTGGTGTCTC |
| **6** | CGCACTTACCCAACCTGGC | GGTCAAAAACGTCAGCCGAG |
| **7** | CGGTGGTGCCCATTTGTTTG | GGCTGGTCAGGATTCTGCTG |
| **8** | CCTTTGCAAAAGCGCAGCAG | GTCGTACTCTCGGAATGCCA |
| **9** | CAAGATTCGCGCGGAGAAGG | AACTCCCAGCCCAGGGTC |
