# Supplementary figures and images for: SOX9 interacts with FOXC1 to activate MYC and regulate CDK7 inhibitor sensitivity in triple-negative breast cancer
Source: Oncogenesis. 2020 May 12;9(5):47. doi: 10.1038/s41389-020-0232-1 (PMC7217837; doi:10.1038/s41389-020-0232-1)

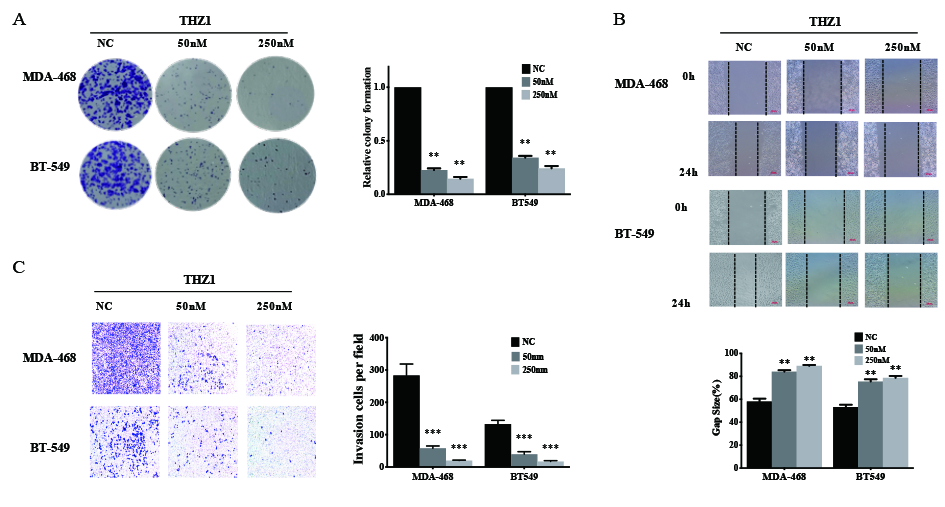

Supplement: Supplementary file 4 — Figure S1 [file 41389_2020_232_MOESM4_ESM.tif]
